# Supplementary material for: BipA exerts temperature-dependent translational control of biofilm-associated colony morphology in Vibrio cholerae
Source: eLife. 2021 Feb 16;10:e60607. doi: 10.7554/eLife.60607 (PMC7886329; doi:10.7554/eLife.60607)
Supplement: Supplementary file 1. [file elife-60607-supp1.docx]

**Supplementary Information**

**BipA exerts temperature-dependent control of biofilm-associated colony morphology in *Vibrio cholerae***

Teresa del Peso Santos^1^, Laura Alvarez^1^, Brandon Sit^2^, Oihane Irazoki^1^, Jonathon Blake^2^, Benjamin R Warner^4^, Alyson R. Warr^2^, Anju Bala^1^, Vladimir Benes^2^, Matthew K. Waldor^2^, Kurt Fredrick^4^ and Felipe Cava^1^

^1^The laboratory for Molecular Infection Medicine Sweden (MIMS), Department of Molecular Biology. Umeå University. Umeå. Sweden.

^2^Howard Hughes Medical Institute, Brigham and Women's Hospital Division of Infectious Diseases and Harvard Medical School Department of Microbiology and Immunobiology, Boston, Massachusetts, USA.

^3^Genomics Core Facility, European Molecular Biology Laboratory (EMBL), Meyerhofstrasse 1, Heidelberg, Germany.

^4^Department of Microbiology, The Ohio State University, Columbus, OH 43210, USA; Center for RNA Biology, The Ohio State University, Columbus, OH 43210, USA.

Correspondence to: [felipe.cava@umu.se](mailto:felipe.cava@umu.se)

Keywords: biofilm, temperature, *Vibrio* *cholerae*, BipA, HapR, translation.

Running title: Temperature-dependent biofilm regulation in *Vibrio cholerae*

**Table of contents:**

Supplementary Figure 1

Supplementary Figure 2

Supplementary Figure 3

Supplementary Figure 4

Supplementary Figure 5

Supplementary Figure 6


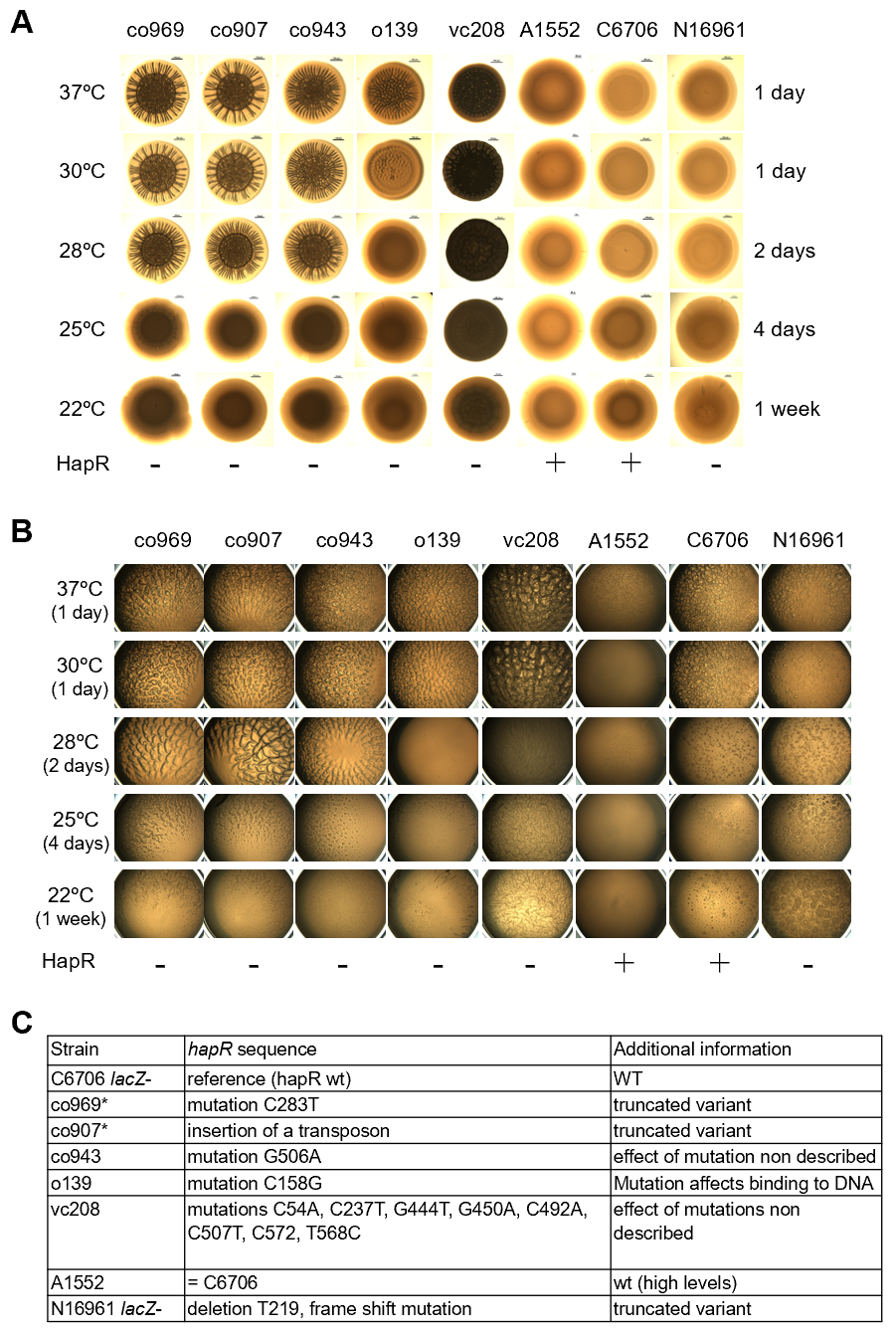


**Supplementary Figure 1.** Effect of temperature on CM and pellicle biofilm of different *V. cholerae* strains.

**A.** Effect of temperature on colony morphology (CM) of different *V. cholerae* strains. Pictures represent the maximum rugosity obtained for different *V. cholerae* strains incubated at different temperatures (37, 30, 28, 25 and 22ºC) during different times. The presence or absence of a functional variant of HapR in these strains is indicated. **B.** Pellicle biofilm formation for different *V. cholerae* strains incubated at different temperatures (37, 30, 28, 25 and 22ºC). The presence or absence of a functional variant of HapR is indicated. **C.** Table indicating the mutations in the *hapR* sequence of the above *V.* *cholerae* strains, which lead to truncated or inactive variants of the protein. Sequencing of *hapR* gene of the strains in panels A and B was performed in this study. To avoid any sequencing errors, sequencing was performed twice from two independent PCR products using primers FCP849 and FCP950 (Supplementary Table 3). The tool <http://web.expasy.org/translate/> allowed to identify which of the changes in the sequence resulted in truncated variants of the protein. The C158G substitution in strain o139 leads to a T53R substitution; T53 has been reported to be involved in binding of HapR to DNA (Singh et al 2014), with a T53A substitution only allowing very weak binding (De Silva et al 2007).


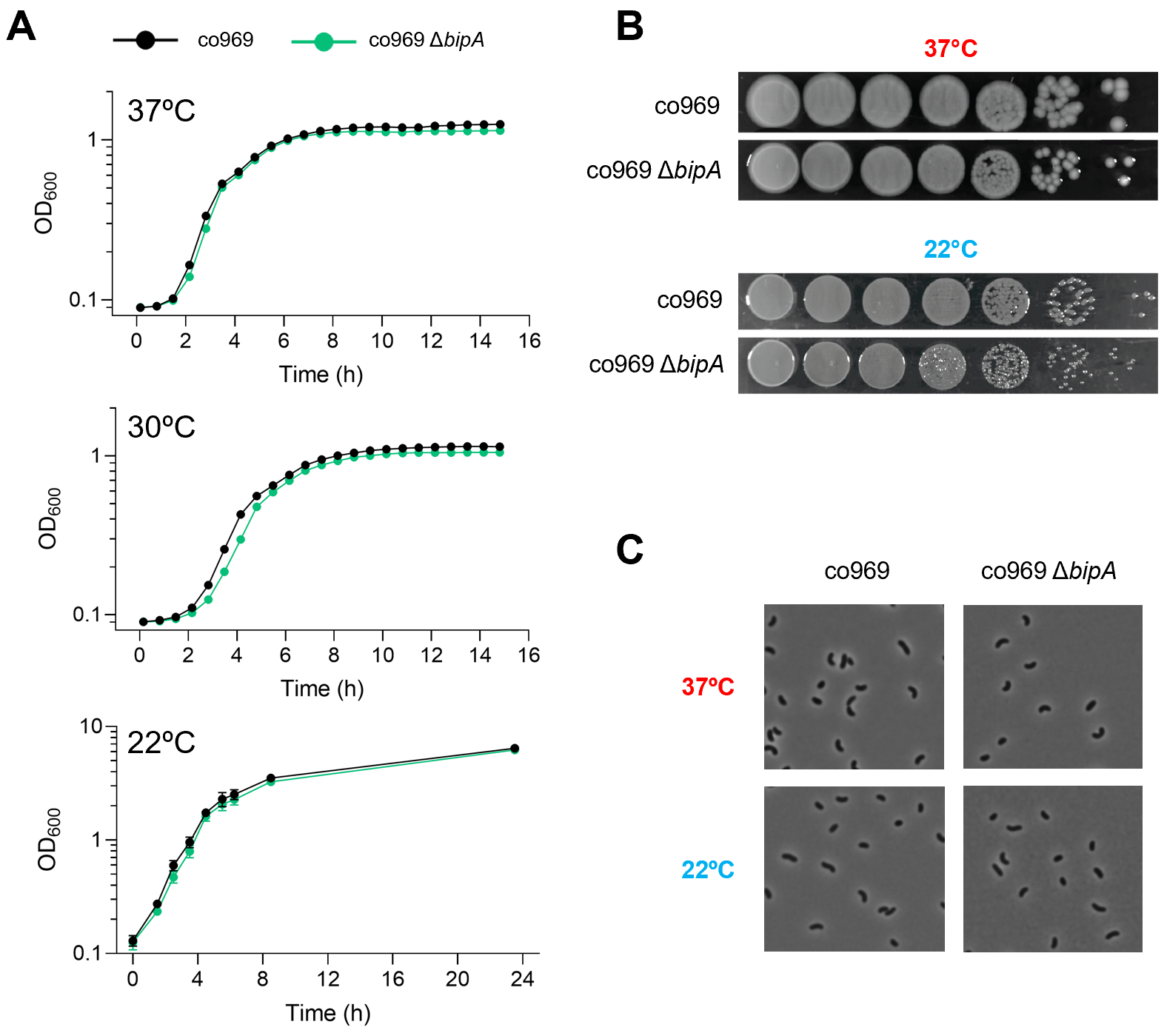


**Supplementary Figure 2.** Growth and cell morphology of *V. cholerae* co969 WT and Δ*bipA* strains at different temperatures. **A.** Growth curves of WT and Δ*bipA* strains at 37, 30 and 22ºC. **B.** Viability of WT and Δ*bipA* strains at 37ºC and 22ºC. **C.** Morphology of co969 and co969 Δ*bipA* grown at 37ºC or 22ºC in stationary phase.


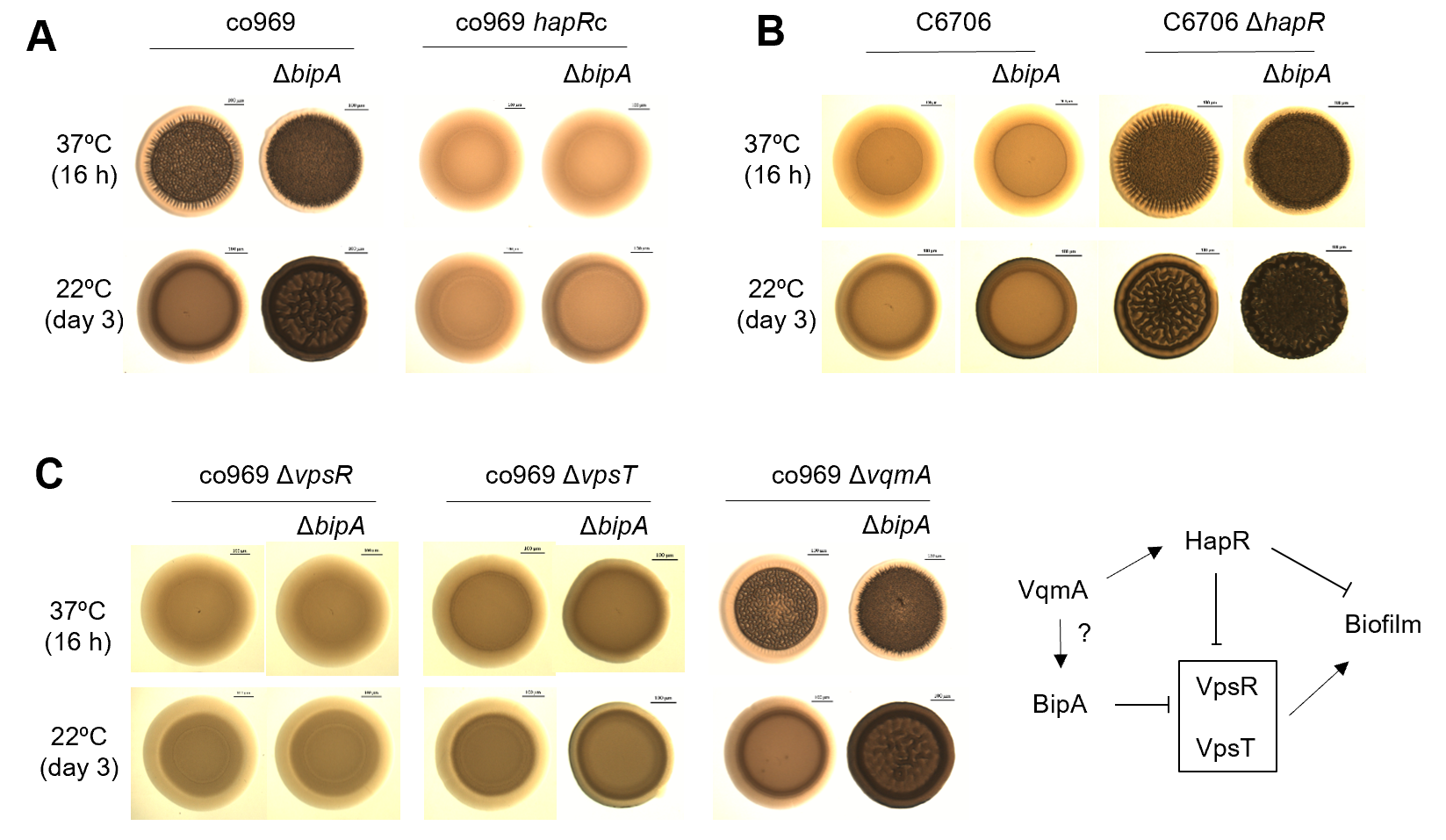


**Supplementary Figure 3.** Interplay of BipA with known biofilm regulators.

**A.** Colony morphology at 37ºC and 22ºC of co969, co969:*hapR*c (with a chromosomal replacement of the mutant *hapR* from co969 with the active *hapR* from C6706, *hapR*c), and their respective Δ*bipA* mutant variants. **B.** Colony morphology at 37ºC and 22ºC of C6706, C6706 Δ*hapR*, and their respective Δ*bipA* mutant variants. **C.** Colony morphology at 37ºC and 22ºC of co969, co969 Δ*vpsR*, co969 Δ*vpsT*, co969 Δ*vqmA*, and their respective Δ*bipA* mutant variants. To the right, schematic showing the impact of these different regulators on biofilm. The incubation times at 37ºC or 22ºC, leading to colonies with a similar number of CFUs/ colony, are indicated for each case.


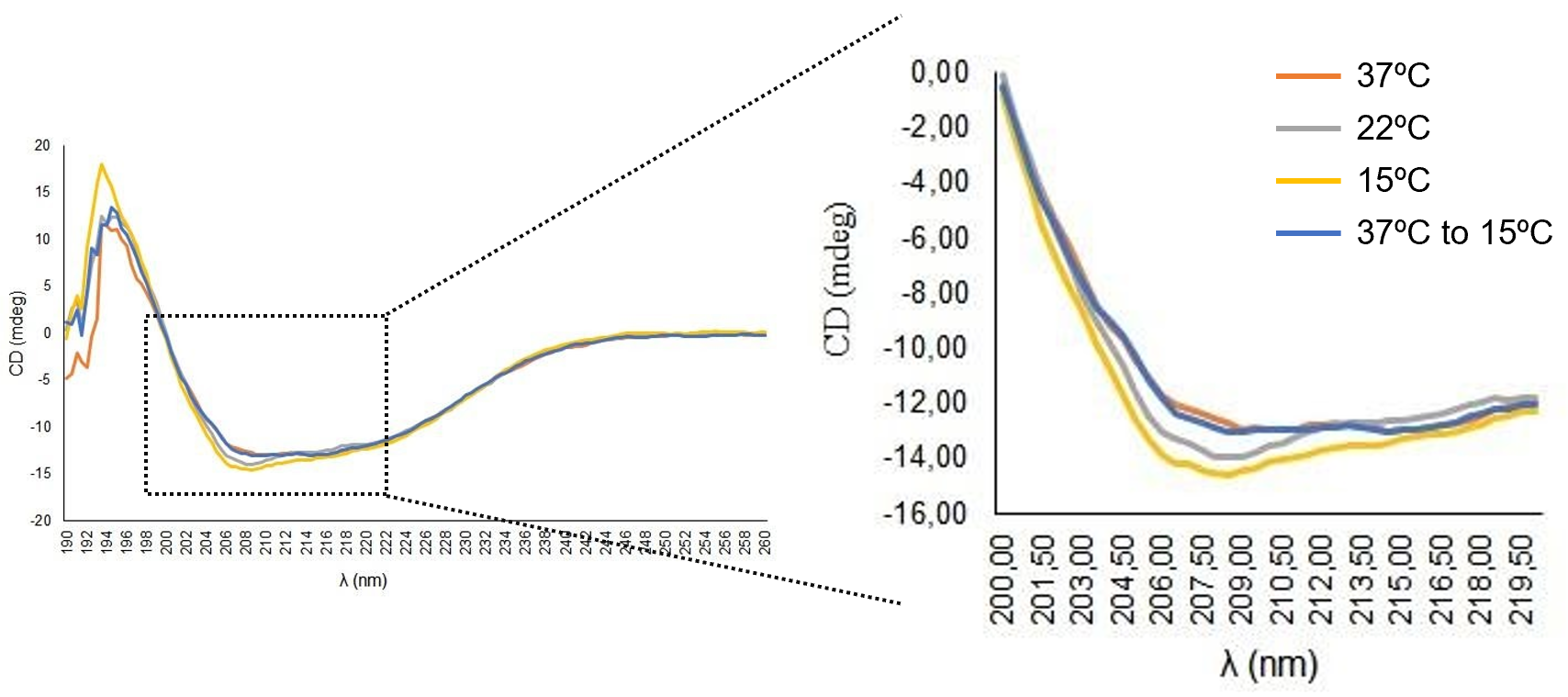


**Supplementary Figure 4.** BipA protein stability at different temperatures. Circular dichroism curve (mdeg represented against wavelength) of purified BipA-His pre-incubated at different temperatures (37ºC, 22ºC or 15ºC) for 30 min, or pre-incubated at 37ºC for 30 min and then sifted to 22ºC and further incubated for 30 min. Boxed graph shows the CD curve between 200 and 220 nm.


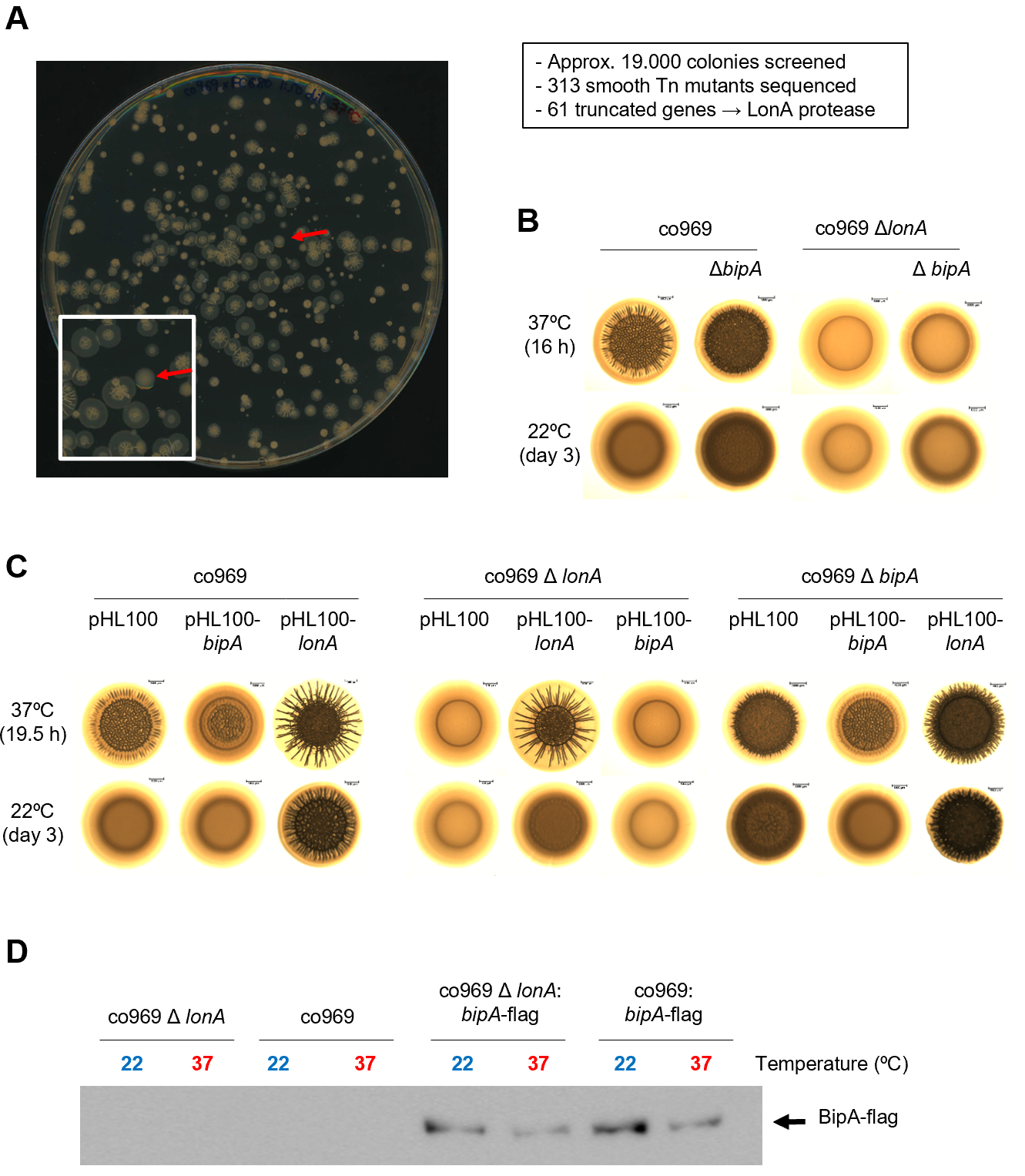


**Supplementary Figure 5.** *V. cholerae* BipA is not degraded by LonA protease.

**A.** Transposon mutagenesis screening for *V. cholerae* co969 mutants with a smooth colony morphology at 37ºC led to identification of LonA. Left, example of a LB (10 g/l NaCl) + Sm200 + Km50 agar plate used for selection of transposon mutants with a smooth colony phenotype at 37ºC (indicated with a red arrow). The number of screened transposon mutants, smooth colonies selected and final number of truncated genes is indicated to the right. **B.** Colony morphology at 37ºC and 22ºC of co969, co969 Δ*lonA* and their respective Δ*bipA* counterpart. **C.** Colony morphology at 37ºC and 22ºC of co969, co969 Δ*bipA* and co969 Δ*lonA* either carrying pHL100-*lonA* or pHL100-*bipA*, to either overexpress *lonA* or *bipA* from the IPTG-inducible P*_lac_* promoter, or the pHL100 empty vector. **D.** Western blot showing the BipA-flag levels from *V. cholerae* co969:*bipA*-flag and co969 Δ*lonA*:*bipA*-flag rugose colonies grown at 37ºC or smooth colonies grown at 22ºC. *V. cholerae* co969 and co969 Δ*lonA* strains were used as a negative control. The gel is a representative of 2 independent experiments, each containing 2 biological replicates, containing each biological replicate 2 colonies pooled together.


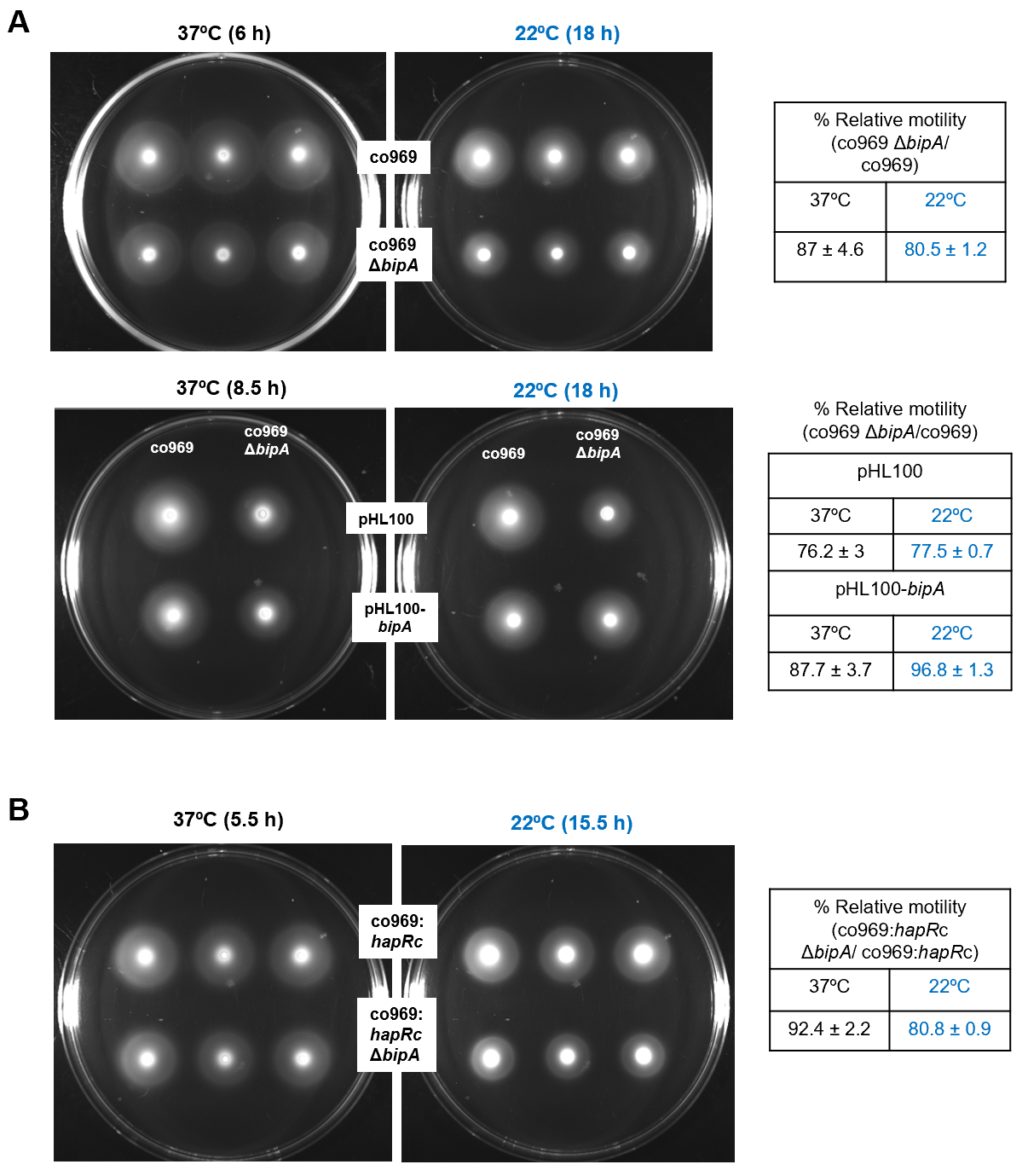


**Supplementary Figure 6.** Effect of BipA on motility.

**A.** Upper panel: motility of co969 and co969 Δ*bipA* on 0.3% agar plates at 37ºC (left) and 22ºC (right) after the indicated times. The relative motility between co969 Δ*bipA* and co969 (in %) at 37ºC or 22ºC is indicated on the right. Lower panel: motility of co969 and co969 Δ*bipA* either carrying pHL100-*bipA*, for overexpression of *bipA* from the IPTG-inducible P*_lac_* promoter, or the empty plasmid pHL100 on 0.3% agar plates with Km (50 µg/ml) and IPTG (isopropyl-β-d-thiogalactosidase, 1 mM) at 37ºC (left) and 22ºC (right) after the indicated times. The relative motility between co969 Δ*bipA* and co969 (in %), either carrying pHL100-*bipA* or pHL100, at 37ºC or 22ºC is indicated on the right. **B.** Motility of co969:*hapR*c and co969:*hapR*c Δ*bipA*, with a chromosomal exchange of the mutant *hapR* from co969 for the active *hapR* of C6706, on 0.3% agar plates at 37ºC (left) and 22ºC (right) after the indicated times. The relative motility between co969:*hapR*c Δ*bipA* and co969:*hapR*c (in %) at 37ºC or 22ºC is indicated to the right. Relative motility values are the average of 2 independent experiments each containing at least 3 biological replicates ± standard deviation.
