# Supplementary material for: BipA exerts temperature-dependent translational control of biofilm-associated colony morphology in Vibrio cholerae
Source: eLife. 2021 Feb 16;10:e60607. doi: 10.7554/eLife.60607 (PMC7886329; doi:10.7554/eLife.60607)
Supplement: Supplementary file 2. [file elife-60607-supp2.docx]

**Supplementary Table 1. Strains used in this study**

| Strain |  | Reference/source |
| --- | --- | --- |
| *Escherichia coli* strains | | |
| DH5α | F^−^,ø80d*lacZ*ΔM15,Δ(*lacZYA-argF*) U169 *deoR*, *recA*1, *endA*1, *hsdR*17 (rk^-^,mk^+^), *phoA*, *supE*44, ʎ^-^, *thi*-1, *gyrA*96, *relA*1 ^-^ | (Hanahan 1983) |
| DH5α λpir | *sup* *E44*, Δ*lacU169* (Φ*lacZ*ΔM15), *recA1*, *endA1*, *hsdR17*, *thi-1*, *gyrA96*, *relA1*, λ*pir* | (Platt et al 2000) |
| SM10 λpir | *thi thr leu tonA lacY supE recA*::RP4-2 Tc::Mu Km ʎ*pir* | (R et al 1983) |
| *Vibrio cholerae* strains | | |
| co969 | new El Tor clinical strain isolated in India (after the re-establishment of O1 dominance after O139; Sm^R^), HapR- | MKW collection |
| co907 | clinical isolate, HapR-, Sm^R^ | MKW collection |
| co943 | clinical isolate, HapR-, Sm^R^ | MKW collection |
| o139 | clinical isolate, HapR-. Sm^R^ | MKW collection |
| vc151 | clinical isolate, HapR-, Sm^R^ | MKW collection |
| vc208 | clinical isolate, HapR-, Sm^R^ | MKW collection |
| A1552 | O1 El Tor, Inaba, Rif^R^ | (Yildiz and Schoolnik 1998) |
| A1552* | O1 El Tor, Inaba, Rif^R^ Sm^R^ | This work |
| C6706 | O1 El Tor strain isolate from Peru; Sm^R^ | (Thelin and Taylor 1996) |
| N16961 | O1 El Tor strain isolate from Bangladesh; Sm^R^ | (Heidelberg et al 2000) |
| co969 Δ*lacZ* | co969, Δ*lacZ* clean deletion mutant; Sm^R^ | This work |
| co969:*hapRc* | co969 with its native *hapR* exchanged for *hapR* of C6706 (*hapR*c); Sm^R^ | This work |
| co969:*hapRc* Δ*bipA* | co969 with its native *hapR* exchanged for *hapR* of C6706 (*hapR*c), Δ*bipA* clean deletion mutant; Sm^R^ | This work |
| co969 Δ*bipA* | co969, Δ*bipA* clean deletion mutant; Sm^R^ | This work |
| co969 Δ*vpsT* | co969, Δ*vpsT* clean deletion mutant; Sm^R^ | This work |
| co969 Δ*vpsT* Δ*bipA* | co969, Δ*vpsT* and Δ*bipA* clean deletion mutants; Sm^R^ | This work |
| co969 Δ*vpsR* | co969, Δ*vpsR* clean deletion mutant; Sm^R^ | This work |
| co969 Δ*vpsR* Δ*bipA* | co969, Δ*vpsR* and Δ*bipA* clean deletion mutants; Sm^R^ | This work |
| co969 Δ*vqmA* | co969, Δ*vqmA* clean deletion mutant; Sm^R^ | This work |
| co969 Δ*vqmA* Δ*bipA* | co969, Δ*vqmA* and Δ*bipA* clean deletion mutants; Sm^R^ | This work |
| co969:*bipA*-flag | co969 with its native *bipA* exchanged for *bipA-*flag; Sm^R^ | This work |
| C6706 Δ*hapR* | C6706, Δ*hapR* clean deletion mutant; Sm^R^ | This work |
| A1552 Δ*hapR* | A1552, Δ*hapR* clean deletion mutant; Sm^R^ | This work |
| co969 Δ*lonA* | co969, Δ*lonA* clean deletion mutant; Sm^R^ | This work |

**Supplementary Table 2. Plasmids used in this study**

| Plasmid | Relevant properties | Reference/source |
| --- | --- | --- |
| pSC189 | Himar 1 suicide transposon vector; Ap^R^, Km^R^ | (Chiang and Rubin 2002) |
| pCB192N | β-galactosidase promoter-probe vector; Ap^R^ | (Kimsey and Waldor 2009) |
| pCB192N-P*vpsR* | pCB192N carrying the P*_vpsR_* promoter; Ap^R^ | This work |
| pCB192N-P*vpsT* | pCB192N carrying the P*_vpsT_* promoter; Ap^R^ | This work |
| pCB192N-P*vpsL* | pCB192N carrying the P*_vpsL_* promoter; Ap^R^ | This work |
| pCB192N-P*vpsU* | pCB192N carrying the P*_vpsU_* promoter; Ap^R^ | This work |
| pCB192N-P*bap1* | pCB192N carrying the P*_bap1_* promoter; Ap^R^ | This work |
| pCB192N-P*gyrA* | pCB192N carrying the P*_gyrA_* promoter; Ap^R^ | This work |
| pCB192N-P*hfq* | pCB192N carrying the P*_hfq_* promoter; Ap^R^ | This work |
| pCB192N-P*mrcA* | pCB192N carrying the P*_mrcA_* promoter; Ap^R^ | This work |
| pCVD442 | oriR6K, *sacB* containing suicide vector for double homologous recombination; Ap^R^ | (Donnenberg and Kaper 1991) |
| pCVD442 Δ*lacZ* | pCVD442-based suicide plasmid for clean deletion of *lacZ*; Ap^R^ | This work |
| pCVD442 Δ*bipA* | pCVD442-based suicide plasmid for clean deletion of *bipA*; Ap^R^ | This work |
| pCVD442-Δ*vpsR* | pCVD442-based suicide plasmid for clean deletion of *vpsR*; Ap^R^ | This work |
| pCVD442-Δ*vpsT* | pCVD442-based suicide plasmid for clean deletion of *vpsT*; Ap^R^ | This work |
| pCVD442-Δ*hapR* | pCVD442-based suicide plasmid for clean deletion of *hapR*; Ap^R^ | This work |
| pCVD442-Δ*vqmA* | pCVD442-based suicide plasmid for clean deletion of *vqmA*; Ap^R^ | This work |
| pCVD442-*hapRc* | pCVD442-based suicide plasmid for chromosomal exchange of *hapR* to *hapR* from C6706 (*hapR*c); Ap^R^ | This work |
| pCVD442 Δ*lonA* | pCVD442-based suicide plasmid for clean deletion of *lonA;* Ap^R^ | This work |
| pHL100 | Replicating plasmid used for expressing genes under control of the IPTG-inducible P*lac* promoter; Km^R^ | (Cava et al 2011) |
| pHL100-*bipA* | *bipA* from *V. cholerae* co969 inserted into BamHI-HindIII of pHL100; Km^R^ | This work |
| pHL100-EC*bipA* | *bipA* from *E. coli* MG1655 K-12 inserted into EcoRI-HindIII of pHL100; Km^R^ | This work |
| pHL100-PP*bipA* | *bipA* from *P. putida* KT2440 K-12 inserted into BamHI-HindIII of pHL100; Km^R^ | This work |
| pHL100-*lonA* | *bipA* from *V. cholerae* co969 inserted into BamHI-HindIII of pHL100; Km^R^ | This work |
| pET22b (+) | Expression vector with T7 promoter and terminator flanking MCS, *pelB* leader sequence for potential periplasmic localization and optional C-terminal His-tag sequence; Amp^R^ | Novagen |
| pET22b-*bipA*-His | *bipA*-His inserted into NdeI-HindIII of pET22b(+) | This work |

**Supplementary Table 3. Primers used in this study**

| FCP | Primer | Sequence (5’ → 3’) | Restriction sites | Use |
| --- | --- | --- | --- | --- |
| FCP  2523 | *vpsA* qPCR fw | GCAAGAAAACTACAACGCAGA |  | qRT-PCR *vpsA* |
| FCP  2524 | *vpsA* qPCR rev | CTCAAAGCCACCACCAAAAC |  |  |
| FCP  2521 | *vpsU* qPCR fw | TATCCAAGTGCGTAGTGCTG |  | qRT-PCR *vpsU* |
| FCP  2522 | *vpsU* qPCR rev | CACTTGCTGGACTGGGTTG |  |  |
| FCP  2525 | *vpsL* qPCR fw | CAATGTGTTGCAAGGAAGTATG |  | qRT-PCR *vpsL* |
| FCP  2526 | *vpsL* qPCR rev | CACTCGCTTCTCCATCTTGT |  |  |
| FCP  2527 | *rbmA* qPCR fw | GCGGAAGTGGATTGTGAGTT |  | qRT-PCR *rbmA* |
| FCP  2528 | *rbmA* qPCR rev | CGCTTTGGCTGGGAAGTAG |  |  |
| FCP  2529 | *rbmC* qPCR fw | GGGAAATCAACAACACAGCA |  | qRT-PCR *rbmC* |
| FCP  2530 | *rbmC* qPCR rev | AAATCCTGAACGAACCGAGA |  |  |
| FCP  2531 | *bap1* qPCR fw | TCTACCAGCGTTTTTCATCCT |  | qRT-PCR *bap1* |
| FCP  2532 | *bap1* qPCR rev | GTCAGCCACATCTTTCTCATC |  |  |
| FCP  2533 | *vpsR* qPCR fw | GCCTTGAAAGCGAGCTGT |  | qRT-PCR *vpsR* |
| FCP  2534 | *vpsR* qPCR rev | CGTCTCCACAGTCCCTTCTT |  |  |
| FCP  2535 | *vpsT* qPCR fw | CAGATTGTTGAAAGAGGCGTTAG |  | qRT-PCR *vpsT* |
| FCP  2536 | *vpsT* qPCR rev | TCGGTCAAAACATCATCAGAA |  |  |
| FCP  2539 | *gyrA* qPCR fw | GTTATCGTGGGTCGTGCTCT |  | qRT-PCR *gyrA* |
| FCP  2540 | *gyrA* qPCR rev | AGGCTTTGTTCCAGTCGTTG |  |  |
| FCP  2541 | *hfq* qPCR fw | TGCAAGGTCAGATCGAATCAT |  | qRT-PCR *hfq* |
| FCP  2542 | *hfq* qPCR rev | TGGTGGCTAACTGGACGAG |  |  |
| FCP  2519 | VC2744 qPCR fw | CGTAGGTGTTATCGGTGTTGG |  | qRT-PCR *bipA* |
| FCP  2520 | VC2744 qPCR rev | CGGTGGCTTGGTCAGTTT |  |  |
| FCP  2537 | *vqmA* qPCR fw | TAAGCAAGGTTTTCGGGATG |  | qRT-PCR *vqmA* |
| FCP  2564 | *vqmA* qPCR fw b | AACGGTGGAAGGGTATGAGG |  |  |
| FCP  799 | *lacZ P1* | CGGTCTAGAGCAAAGGCGTTATTG | XbaI | *lacZ* clean deletion mutant  (pCVD442 Δ*lacZ*) |
| FCP  800 | *lacZ P2* | TTTTTTGCGGCCGCTTTTTTCTCGGCAGGCAAGCGGCTATC |  |  |
| FCP  801 | *lacZ P3* | AAAAAAGCGGCCGCAAAAAACTGAATGTTGAGGTGATGCTGTG |  |  |
| FCP  802 | *lacZ P4* | CGGTCTAGAGAGCAAAAATTCAGGGTG | XbaI |  |
| FCP  803 | *lacZ check fw* | GGTGAGTGGTTCACAGAATCGGTG |  |  |
| FCP  804 | *lacZ check rev* | CTCAGTTGGCTCTTGCTTTGGCAAC |  |  |
| FCP  805 | *lacZ check-2 fw* | GCTGGATCGCACGTTGGCATG |  |  |
| FCP  806 | *lacZ check-2 rev* | CGGCTTGCGGTAGAGATACACATC |  |  |
| FCP  1533 | VC2744-P1 | GGCAGAGCTCTGGTGCTGGAATGTTTG | SacI | *bipA* clean deletion mutant  (pCVD442 Δ*bipA*) |
| FCP  1534 | VC2744-P2 | TTTTTTGCGGCCGCTTTTTTCGGGGTTTGCTTCACTTTTTCATTG |  |  |
| FCP  1535 | VC2744-P3 | AAAAAAGCGGCCGCAAAAAAATCACTGACTTTCGTTTGGTAAG |  |  |
| FCP  1536 | VC2744-P4 | CGCGAGCTCGCTGACCAATATTTGGCAG | SacI |  |
| FCP  1537 | VC2744-check | GCCGCCAATCATTATGTCTC |  |  |
| FCP  2437 | VC2744 check rev | TTGGCACATCAATACACTGA |  |  |
| FCP  1317 | VCA0952 P1 | GGATGTCGACGCTATTGATATTC | SalI | *vpsT* clean deletion mutant  (pCVD442 Δ*vpsT*) |
| FCP  1318 | VCA0952 P2 | TTTTTTGCGGCCGCTTTTTTCACATCAAGGCTAACATG |  |  |
| FCP  1319 | VCA0952 P3 | AAAAAAGCGGCCGCAAAAAAGTCAATTCTTAATTCGTTGTG |  |  |
| FCP  1320 | VCA0952 P4 | GGGTCGACTGCCGTAGTCGGTATC | SalI |  |
| FCP  1483 | VCA0952 check | CCAAGAATACATTCTCCATTATC |  |  |
| FCP  2434 | VCA0952 check rev | GGGTGGACGCTCTGGCACT |  |  |
| FCP  1307 | VC0665 P1 | GGGTCTAGATATTGGTATTGATCGTC | XbaI | *vpsR* clean deletion mutant  (pCVD442 Δ*vpsR*) |
| FCP  1308 | VC0665 P2 | TTTTTTGCGGCCGCTTTTTTCAGGGTTCATAGGTACCTC |  |  |
| FCP  1309 | VC0665 P3 | AAAAAAGCGGCCGCAAAAAACGCGTGCAACCATGTATC |  |  |
| FCP  1310 | VC0665 P4 | GCCATCTAGAGCCTCCAAGCAAAGTC | XbaI |  |
| FCP  1480 | VC0665 check | GGATGAGTCTCAGCTCGATC |  |  |
| FCP  2436 | VC0665 check rev | CGCACTTTACGCTTCCACACT |  |  |
| FCP  845 | *hapR* P1 | AAAATCTAGACTTCACGACGGGTTG | XbaI | *hapR* clean deletion mutant  (pCVD442 Δ*hapR*) |
| FCP  846 | *hapR* P2 | TTTTTTGCGGCCGCTTTTTTCATAGGGGTATATCCTTG |  |  |
| FCP  847 | *hapR* P3 | AAAAAAGCGGCCGCAAAAAAGTTTCTTGGGCAGCAC |  |  |
| FCP  848 | *hapR* P4 | AAAATCTAGACCATGTGCGGTGATC | XbaI |  |
| FCP  849 | *hapR* checkfw | GCACCATTACACTCATAGG |  |  |
| FCP  850 | *hapR* checkrv | CCTGCAACACCAAGTCG |  |  |
| FCP  845 | *hapR* P1 | AAAATCTAGACTTCACGACGGGTTG |  | Chromosomal Exchange by *hapR* from C6706  (pCVD442 *hapR*c) |
| FCP  848 | *hapR* P4 | AAAATCTAGACCATGTGCGGTGATC |  |  |
| FCP  2512 | VCA1078 P1 | CACGGTCTAGACAGGAACCTCTTGGTC | XbaI | *vqmA* clean deletion mutant  (pCVD442 Δ*vqmA*) |
| FCP  2517 | VCA1078 P2 | TTTTTTGCGGCCGCTTTTTTGGGGATCGTGTGATCGCTC |  |  |
| FCP  2518 | VCA1078 P3 | AAAAAAGCGGCCGCAAAAAAAGAGCAGATTTCTTTATTC |  |  |
| FCP  2515 | VCA1078 P4 | GCGATTCTAGATGCGACGGTAGATGAACAC | XbaI |  |
| FCP  2516 | VCA1078 check | GGTATGAGGCCAAACTACGCAG |  |  |
| FCP  3244 | VC1920 P1 | GGTTTGATtCtAGAGTTCATTGGTCGTCTG | XbaI | *lonA* clean deletion mutant  (pCVD442 Δ*lonA*) |
| FCP  3245 | VC1920 P2 | TTTTTTGCGGCCGCTTTTTTATTTTTCTCTCTTCCGCTTTG |  |  |
| FCP  3246 | VC1920 P3 | AAAAAAGCGGCCGCAAAAAAGCAAAAATAAGTAAATCTTTAC |  |  |
| FCP  3247 | VC1920 P4 | CCTCAGATCTAGAAATGCGCATCAGTAGGATG | XbaI |  |
| FCP  3248 | VC1920 check | GCGATGACGGTGGTGCGCTCTC |  |  |
| FCP  1204 | P*vpsR* HindIII fw | CCGAAGCTTACCAATACTCACACTATC | HindIII | P*_vpsR_*, translational β-galactosidase reporter |
| FCP  1206 | P*vpsR* EcoRI rev | CCATACGGGAATTCCCTTTCATGAACCTATATTC | EcoRI |  |
| FCP  1208 | P*vpsT* HindIII fw | GCATGAAGCTTAAGTGCTTTATCGCAC | HindIII | P*_vpsT_*, translational β-galactosidase reporter |
| FCP  1210 | P*vpsT* EcoRI rev | CGTTTGGAATTCCCTTTCATTTCACCCCTCCTAAC | EcoRI |  |
| FCP809 | P*vpsL* HindIII fw | GGCTAAGCTTCTTGTGTACATAGAGCAG | HindIII | P*_vpsL_*, translational β-galactosidase reporter |
| FCP5381 | P*vpsL* EcoRI rev | GTGCTTGAATTCCCTTTCATTAGACGCTCCTAAC | EcoRI |  |
| FCP5382 | P*vpsU* BglII fw | CCGCTCAGATCTAAAATTGGTTATCCCATC | BglII | P*_vpsU_*, translational β-galactosidase reporter |
| FCP5383 | P*vpsU* EcoRI rev | CCGAGAAAGAATTCCCTTTCATACCCTAACTAC | EcoRI |  |
| FCP779 | P*bap1* HindIII fw | GTCCGAAGCTTTGCTGAGTAAGACTC | HindIII | P*_bap1_*, translational β-galactosidase reporter |
| FCP5379 | P*bap1* EcoRI rev | CCACGTTGAATTCCCTTTCATGGCTTGACCTTC | EcoRI |  |
| FCP3025 | P*gyrA* HindIII fw | CCTTGAAGCTTTCTTGTATCGCCTGTG | HindIII | P*_gyrA_*, translational β-galactosidase reporter |
| FCP3026 | P*gyrA* EcoRI rev | CCTTAGAATTCCCTTTCATAGAGCCATTATCCCTC | EcoRI |  |
| FCP5388 | P*hfq* HindIII fw | GGAAGCAAAGCTTAATGTGATCGCTTTTGCGT | HindIII | P*_hfq_*, translational β-galactosidase reporter |
| FCP5389 | P*hfq* EcoRI rev | GTAGAGATGAATTCCCTTTCATTTTGTTTTTCCTTATTTTGTC | EcoRI |  |
| FCP5390 | P*mrcA* HindIII fw | CCGATTTAAGCTTGAGGCTAAGCTCCAG | HindIII | P*_mrcA_*, translational β-galactosidase reporter |
| FCP5391 | P*mrcA* EcoRI rev | CCCTGTTTGAATTCCCTTTCATTAATGCAAACCCTTGTAC | EcoRI |  |
| FCP  1687 | BamHI-RBS-VC2744 fw | GTGAAGGATCCAGGAGGACCCCGATGACCACTCCGCAAATTG | BamHI | Overexpression of *V.* *cholerae* co969 *bipA* from pHL100 |
| FCP  1688 | HindIII-VC2744 rev | CGAAAGTAAGCTTTTACTTCGCATCACGAGAAGC | HindIII |  |
| FCP  3101 | EcoRI-RBS-ECbipA fw | GCTATTTCTGAATTCAGGAGGAAAGTTGTGATCGAAAAATTG | EcoRI | Overexpression of *E.* *coli* MG1655 K-12 *bipA* from pHL100 |
| FCP  3102 | ECbipA-HindIII | GGGAAAAGCTTTTAATCGTCTTTCGGTG | HindIII |  |
| FCP  3103 | BamHI-RBS-PPbipA fw | GGATCCGGATCCAGGAGGACACTTGTGATCGAAAATCTG | BamHI | Overexpression of *P.* *putida* KT2440 *bipA* from pHL100 |
| FCP  3104 | PPbipA-HindIII rev | CCTGAaagcttTCAGTTCTTGGCTTTCTTG | HindIII |  |
| FCP  3249 | VC1920 SacI fw  (SacI-RBS-VC1920 fw) | GGTGCAAGAGCTCAGGAGGAAAAATATGAACTTGGAGCGTTC | SacI | Overexpression of *lonA* from pHL100 |
| FCP  3250 | VC1920 BamHI rev | CCACTTAGGATCCCTATGCATCACTCTTTTTGCTC | BamHI |  |
| FCP  2511 | NdeI-VC2744 fw | CAAACCCATATGACCACTCCGCAAATTG | NdeI | Purification of BipA-His (from pET22b-*bipA*-His) |
| FCP  2510 | VC2744-His-stop-HindIII-rev | GATGACTTAAGCTTTTAGTGGTGGTGGTGGTGGTGCTTCGCATCACGAGAAG | HindIII |  |
| FCP  1822 | VC2744-flag P1 | CGTCTTCTAGACCATTCATTTGACCACTAC | XbaI | *bipA*-flag chromosomal phusion |
| FCP  1823 | VC2744-flag P2 | TTACTTATCGTCATCGTCCTTGTAGTCCTTCGCATCACGAGAAGCAC |  |  |
| FCP  1824 | VC2744-flag P3 | GACTACAAGGACGATGACGATAAGTAATCACTGACTTTCGTTTG |  |  |
| FCP  1825 | VC2744-flag P4 | CCTGTTCTAGATAGCCCGAGCTCGCTGAC | XbaI |  |
| FCP  2437 | VC2744 check rev | TTGGCACATCAATACACTGA |  |  |
| FCP  1461 | flag fw | GACTACAAGGACGATGACGATAAG |  | Checking flag chromosomal fusions |
| FCP  718 | CVD442 F | CCAGCCCTCCTGTTTGAAGATG |  | Checking and sequencing inserts into pCVD442 |
| FCP  719 | CVD442 R | ACTGAGAAGCCCTTAGAGCC |  |  |
| FCP  224 | YPR505 | GCCGACATCATAACGGTTCTGG |  | Checking and sequencing inserts into pHL100 |
| FCP  643 | Pbad33-BKD | CAGGCTGAAAATCTTCTCTCATCC |  |  |
| FCP  39 | T7 forward | TAATACGACTCACTATAGGG |  | Checking and sequencing inserts into pET22b(+) |
| FCP  40 | T7 terminator primer | CTAGTTATTGCTCAGCGGTG |  |  |

**Supplementary references**

Cava F, de Pedro MA, Lam H, Davis BM, Waldor MK (2011). Distinct pathways for modification of the bacterial cell wall by non-canonical D-amino acids. *EMBO J* **30:** 3442-3453.

Chiang SL, Rubin EJ (2002). Construction of a mariner-based transposon for epitope-tagging and genomic targeting. *Gene* **296:** 179-185.

De Silva RS, Kovacikova G, Lin W, Taylor RK, Skorupski K, Kull FJ (2007). Crystal structure of the Vibrio cholerae quorum-sensing regulatory protein HapR. *Journal of bacteriology* **189:** 5683-5691.

Donnenberg MS, Kaper JB (1991). Construction of an eae deletion mutant of enteropathogenic Escherichia coli by using a positive-selection suicide vector. *Infection and immunity* **59:** 4310-4317.

Hanahan D (1983). Studies on transformation of Escherichia coli with plasmids. *J Mol Biol* **166:** 557-580.

Heidelberg JF, Eisen JA, Nelson WC, Clayton RA, Gwinn ML, Dodson RJ *et al* (2000). DNA sequence of both chromosomes of the cholera pathogen Vibrio cholerae. *Nature* **406:** 477-483.

Kimsey HH, Waldor MK (2009). Vibrio cholerae LexA coordinates CTX prophage gene expression. *Journal of bacteriology* **191:** 6788-6795.

Platt R, Drescher C, Park S-K, Phillips GJ (2000). Genetic System for Reversible Integration of DNA Constructs and lacZ Gene Fusions into the Escherichia coli Chromosome. *Plasmid* **43:** 12-23.

R S, U P, A P (1983). A broad host range mobilization system for in vivo genetic engineering: transposon mutagenesis in gram negative bacteria. *Biotechnology* **1:** 784-791.

Singh NS, Kachhap S, Singh R, Mishra RC, Singh B, Raychaudhuri S (2014). The length of glycine-rich linker in DNA-binding domain is critical for optimal functioning of quorum-sensing master regulatory protein HapR. *Molecular Genetics and Genomics* **289:** 1171-1182.

Thelin KH, Taylor RK (1996). Toxin-coregulated pilus, but not mannose-sensitive hemagglutinin, is required for colonization by Vibrio cholerae O1 El Tor biotype and O139 strains. *Infection and immunity* **64:** 2853-2856.

Yildiz FH, Schoolnik GK (1998). Role of rpoS in stress survival and virulence of Vibrio cholerae. *J Bacteriol* **180:** 773-784.
